# A247 THE IMPACT OF BIOLOGICS ON MATERNAL AND NEONATAL OUTCOMES IN PREGNANT PATIENTS WITH INFLAMMATORY BOWEL DISEASE AND THEIR ASSOCIATION WITH THERAPEUTIC DRUG LEVELS

**DOI:** 10.1093/jcag/gwad061.247

**Published:** 2024-02-14

**Authors:** H Nabavian, S Eisen, K O'Connor, V Srikanth, V Huang

**Affiliations:** Medicine, University of Toronto, Toronto, ON, Canada; Medicine, University of Toronto, Toronto, ON, Canada; Mount Sinai Health System, Toronto, ON, Canada; Mount Sinai Health System, Toronto, ON, Canada; Mount Sinai Health System, Toronto, ON, Canada

## Abstract

**Background:**

Biologic therapies are at the backbone of managing moderate to severe IBD and maintaining clinical remission. The data on the effects of biologic therapy on maternal and neonatal outcomes continues to be limited. To our knowledge, there have been no previous studies specifically investigating whether biologic therapeutic drug levels in pregnant patients are related to maternal and neonatal outcomes.

**Aims:**

The objective of this study is to determine whether biologic use in pregnancy is associated with adverse maternal and fetal outcomes, and if those outcomes correlate to specific therapeutic drug levels.

**Methods:**

In this study, we retrospectively evaluate patients at a high-volume pregnancy and inflammatory bowel disease centre at Mount Sinai Hospital in Toronto between January 1st, 2017 to December 31st, 2022. Primary outcomes include maternal complications, such as induction of labour, cesarean section, peripartum infections, placental abruption, preterm labour and pre-eclampsia. Secondary outcomes include neonatal complications, such as preterm birth, low birth weight (ampersand:003C2500g), congenital anomalies and the presence of neonatal infection. These outcomes will be compared to therapeutic drug levels measured in preconception, during pregnancy and post-partum.

**Results:**

A total of 312 patients were included in our study, of which 152 were on biologic treatment for IBD. Of the biologics population, the majority of patients are on Remicade, followed by Humira and Stelara (Table 1). Preliminary data analysis reveals that there was no significant difference in the rate of miscarriages (10% vs. 6%, P= 0.1), cesarean section (44% vs. 38%, P=0.3) and preterm deliveries (12% vs. 14%, P=0.7) between the biologics vs. non-biologics patient groups. Next round of analysis will include secondary outcomes and therapeutic drug levels.

**Conclusions:**

Through this study, we hope to add to the literature on the possible safety of maintaining biologic therapy during pregnancy in those with inflammatory bowel disease. The data on proactive therapeutic drug monitoring in pregnant patients may bring to light the importance of its inclusion in clinical decision-making algorithms.

Breakdown of therapies in the biologic patient group

**Funding Agencies:**

None

**Table:** 

IBD-Related Biologic (n=152)	Number of patients
Remicade	67
Humira (Originator)	47
Adalimumab (Biosimilar)	2
Vedolizumab (Entyvio)	17
Ustekinumab (Stelara)	18
Golimumab (Simponi)	1

